# Challenges of Treatment-Resistant Bipolar Depression in the Elderly: A Case Study of Successful Modafinil Augmentation

**DOI:** 10.7759/cureus.96094

**Published:** 2025-11-04

**Authors:** Arinze Mbanusi, Muhammad-Kazim Kanani

**Affiliations:** 1 Mental Health Services for Older People, Lanchester Road Hospital, Tees, Esk and Wear Valleys NHS Foundation Trust, Durham, GBR

**Keywords:** bipolar depression, elderly psychiatry, modafinil, psychopharmacology, treatment-resistant depression

## Abstract

Treatment-resistant bipolar depression (TRBD) poses a significant challenge, especially in older adults, where comorbidities and medication tolerability limit therapeutic options. Modafinil, a wakefulness-promoting agent, has shown promise as an adjunctive treatment in mood disorders. We report the case of a 74-year-old man with long-standing bipolar disorder and multiple comorbidities, including chronic kidney disease (CKD) and cerebellar ataxia, who presented with persistent depressive symptoms despite multiple pharmacological trials. Conventional treatments, including lithium, antipsychotics, and antidepressants, were either ineffective or poorly tolerated. Modafinil was introduced as an augmentative strategy, starting at 100 mg daily and titrated to 200 mg. Over 16 weeks, the patient demonstrated marked improvement in mood, motivation, and wakefulness, with his Montgomery-Åsberg Depression Rating Scale (MADRS) score reduced by 23 points (70%) from 29 to 6. No adverse effects or mood destabilization were observed. This case highlights the potential utility of modafinil in TRBD, particularly in elderly patients with limited pharmacological options. Its broad neurochemical activity and tolerability profile make it a candidate for augmentation in selected cases. While caution is warranted due to potential risks, this report supports further exploration of modafinil in adult populations with TRBD. Modafinil may be a safe and effective adjunct in treatment-resistant bipolar depression, especially when conventional therapies are contraindicated. Its use may extend beyond elderly populations to adults with similar clinical challenges.

## Introduction

Bipolar depression presents a significant treatment challenge, often overshadowing the manic and hypomanic phases of the disorder. Despite advancements in pharmacotherapy, a substantial proportion of patients experience persistent depressive symptoms even after multiple medication trials [1]. Treatment-resistant bipolar depression (TRBD) is particularly pronounced in older adults, where standard mood stabilizers are often limited by adverse effects and comorbid conditions such as chronic kidney disease (CKD), cardiovascular disease, and cognitive impairment [2,3].

Lithium, a cornerstone in bipolar disorder management, is known for its mood-stabilizing efficacy but is associated with nephrotoxicity, restricting its long-term use in elderly individuals [4]. Similarly, antipsychotics and antidepressants, although commonly used, often fail to provide sustained relief and can contribute to metabolic, cognitive, and cardiovascular side effects. The search for alternative strategies has led to an increasing interest in adjunctive therapies, particularly those targeting neurotransmitter systems beyond traditional mood stabilizers.

Modafinil, a wakefulness-promoting agent primarily approved for narcolepsy, has emerged as a potential option for TRBD. Its mechanism, involving the modulation of dopamine, serotonin, catecholamines, histamine, glutamate, and GABA, suggests a broader neurochemical impact that could benefit patients with refractory mood symptoms [5]. Several studies, including a meta-analysis of randomized controlled trials [2], suggest that modafinil enhances motivation, alertness, and mood while maintaining a relatively low risk of inducing mania. However, data on its use in the elderly population remains limited.

This case report presents the successful augmentation of modafinil in an elderly patient with treatment-resistant bipolar depression. The patient had exhausted conventional pharmacological options, necessitating an alternative approach. His response to modafinil was not only clinically significant but also provided insight into the viability of this treatment strategy in psychiatric care.

## Case presentation

A 74-year-old Caucasian man with a long-standing history of bipolar disorder and multiple comorbidities, including hypertension, pulmonary fibrosis, cerebellar ataxia, and chronic kidney disease (CKD stage 3b, eGFR: 40 mL/min/1.73 m²), was referred for ongoing management of persistent depressive symptoms. His psychiatric history dated back to 1995, initially presenting with depressive symptoms before being diagnosed with bipolar disorder following a manic episode two years later in 1997. He also had a history of harmful alcohol use (up to 50 units/week), although he had stopped this habit in 2013, seven years before our involvement.

Initially treated with lithium and venlafaxine, he remained stable for several years. Lithium was discontinued in 2006 after approximately nine years of use, due to declining renal function. He continued venlafaxine, but in the following years, he experienced mood fluctuations, recurrent hypomanic episodes, and worsening depressive symptoms. He also reported cognitive concerns and gait instability, which were secondary to cerebellar ataxia.

In 2013, he had a severe manic relapse necessitating inpatient admission. Subsequently diagnosed with bipolar mania with psychosis, he was stabilized on olanzapine. Following discharge, he experienced a depressive relapse, and venlafaxine was initiated and titrated accordingly. Despite trials of lamotrigine, fluoxetine, aripiprazole, and quetiapine, his depressive symptoms persisted. Lithium was reintroduced in 2015 but discontinued due to worsening renal function and poor therapeutic response. Lurasidone was introduced later but caused significant gastrointestinal side effects and was stopped.

During our care (elderly psychiatric clinic), beginning in 2020, venlafaxine dosage was reduced due to urinary symptoms, and he was under the care of urology. Quetiapine was initiated but discontinued due to oversedation. Aripiprazole was ineffective for depressive symptoms. Olanzapine was reintroduced later, as he had previously shown a partial response, but dosing was limited to 10 mg due to postural hypotension, believed to be compounded by cerebellar ataxia. Fluoxetine was also trialed with lamotrigine, but no significant improvement was observed. Despite these numerous adjustments (Table 1), he continued to report marked anhedonia, hypersomnia, and low motivation. Potential organic causes of depression were considered, but no significant functional decline was observed to suggest an underlying neurodegenerative condition. An MRI performed in 2011 revealed generalized cerebral and cerebellar atrophy without acute pathology. During our current care, a head CT scan was requested in 2021, which showed no new or acute changes. His Addenbrooke’s Cognitive Examination III (ACE-III) score was 85/100, excluding major neurocognitive disorder [6].

**Table 1:** Medication history, renal trends, and rationale eGFR: estimated glomerular filtration rate (mL/min/1.73 m²), CKD: chronic kidney disease, GI: gastrointestinal

Medication and dose	Year	eGFR (mL/min/1.73 m²)	Creatinine (umol/L)	Rationale
Lithium 600 mg, venlafaxine 75 mg	1997-2006	Normal at initiation	Normal at initiation	Initial stability: lithium stopped due to CKD
Venlafaxine 112.5 mg	2006-2011	55	117	Maintained partial response
Sertraline 100 mg	2011-2013	79	87	Trial for depressive symptoms
Venlafaxine 75 mg, lamotrigine 150 mg, olanzapine 10 mg	2013-2014	62	106	Post-mania relapse; depressive phase
Fluoxetine 20 mg, lamotrigine 300 mg, olanzapine 5 mg	2014	52	122	Olanzapine stopped due to hypotension
Lithium 400 mg, venlafaxine 225 mg, lamotrigine 300 mg	2015	52	121	Lithium re-trial: stopped due to renal decline and poor response
Aripiprazole 5 mg, venlafaxine 300 mg, lamotrigine 300 mg	2016-2020	39	156	Aripiprazole stopped due to lack of efficacy for depression
Quetiapine 200 mg, venlafaxine 225 mg, lamotrigine 300 mg	2020-2021	47	147	Quetiapine offered under current care; stopped due to oversedation
Olanzapine 10 mg, venlafaxine 75 mg, lamotrigine 300 mg	2021-2024	42	143	Olanzapine reoffered based on prior positive response; dose increase limited by postural hypotension
Lurasidone 18.5 mg, venlafaxine 75 mg, lamotrigine 300 mg	2024	41	145	Lurasidone discontinued due to GI side effects
Modafinil 200 mg, venlafaxine 75 mg, lamotrigine 300 mg	2024-2025	40	147	Modafinil adjunct for persistent depressive symptoms

Given persistent depressive symptoms and limited pharmacological options, the team discussed augmentative strategies. Electroconvulsive therapy (ECT) was considered, but not pursued, as the anesthetic team deemed him unsuitable for general anesthesia due to comorbid pulmonary fibrosis. As alternatives, the team explored pharmacological augmentation with either modafinil or cariprazine. Cariprazine, an atypical antipsychotic and partial agonist at D2/D3 and 5-HT1A receptors with evidence in bipolar depression [7], was offered, but the patient preferred modafinil.

By September 2024, he was started on modafinil 100 mg in the morning. After four weeks, there was mild improvement in mood and energy, prompting an increase to 100 mg twice daily. Following dose adjustment, his Montgomery-Åsberg Depression Rating Scale (MADRS) score improved from 29/60 to 6/60, a 23-point (79%) reduction over a 16-week period [8].

## Discussion

TRBD is characterized by the persistence of depressive symptoms despite adequate trials of standard treatments. While definitions vary, a common criterion is the failure to achieve symptom improvement after at least two therapeutic interventions [2]. It remains a significant challenge in psychiatric management, particularly in the older population, where medication tolerability is a primary concern. Standard treatment strategies include mood stabilizers, atypical antipsychotics, and antidepressants; however, these are often limited by adverse effects, drug interactions, and patient-specific contraindications [3].

Lithium remains highly effective but poses risks for older adults with renal impairment [4]. Lamotrigine, which was already part of this patient’s regimen, has demonstrated efficacy in the treatment of bipolar depression as supported by a recent systematic review [9]. Lurasidone, evidenced by clinical trials for bipolar depression with minimal metabolic side effects [10], was also trialed but was not tolerated due to gastrointestinal side effects.

Modafinil has gained attention as a novel adjunctive treatment for bipolar depression due to its wakefulness-promoting effects and ability to modulate multiple neurotransmitter pathways [4]. Reports from a meta-analysis suggested that modafinil at a dose of 174.2 mg was well tolerated, enhanced mood and motivation, and showed no significant difference in mania induction between placebo and treatment groups [11]. A case report of an elderly woman with bipolar depression also documented improvement in mood and functional engagement [12]. However, its use in the elderly warrants caution due to potential risks such as psychosis, mania, agitation, cardiovascular effects, orofacial dyskinesia, and insomnia.

This patient’s positive response, alongside careful monitoring, supports modafinil’s role in highly selected elderly TRBD cases.

## Conclusions

In this case, modafinil provided a well-tolerated augmentation strategy, leading to significant improvements in mood, wakefulness, and daily functioning. Notably, no adverse effects or mood destabilization were observed. This suggests that modafinil may be a viable adjunct in elderly patients with TRBD, particularly when standard treatments are contraindicated. Further studies, especially randomized controlled trials, are needed to clarify the efficacy, tolerability, and safety of modafinil in elderly populations with bipolar depression. While this case involved an elderly patient, modafinil may also be trialed in adults with treatment-resistant bipolar depression where conventional options are limited.

## References

[1] Baldessarini RJ, Vázquez GH, Tondo L (2020). Bipolar depression: a major unsolved challenge. Int J Bipolar Disord.

[2] Elsayed OH, Ercis M, Pahwa M, Singh B (2022). Treatment-resistant bipolar depression: therapeutic trends, challenges and future directions. Neuropsychiatr Dis Treat.

[3] Shobassy A (2021). Elderly bipolar disorder. Curr Psychiatry Rep.

[4] Ulrichsen A, Hampsey E, Taylor RH, Gadelrab R, Strawbridge R, Young AH (2023). Comparing measurements of lithium treatment efficacy in people with bipolar disorder: systematic review and meta-analysis. BJPsych Open.

[5] Gerrard P, Malcolm R (2007). Mechanisms of modafinil: a review of current research. Neuropsychiatr Dis Treat.

[6] Hsieh S, Schubert S, Hoon C, Mioshi E, Hodges JR (2013). Validation of the Addenbrooke's Cognitive Examination III in frontotemporal dementia and Alzheimer's disease. Dement Geriatr Cogn Disord.

[7] Earley W, Burgess MV, Rekeda L (2019). Cariprazine treatment of bipolar depression: a randomized double-blind placebo-controlled phase 3 study. Am J Psychiatry.

[8] Montgomery SA, Asberg M (1979). A new depression scale designed to be sensitive to change. Br J Psychiatry.

[9] Haenen N, Kamperman AM, Prodan A, Nolen WA, Boks MP, Wesseloo R (2024). The efficacy of lamotrigine in bipolar disorder: a systematic review and meta-analysis. Bipolar Disord.

[10] Bawa R, Scarff JR (2015). Lurasidone: a new treatment option for bipolar depression-a review. Innov Clin Neurosci.

[11] Nunez NA, Singh B, Romo-Nava F (2020). Efficacy and tolerability of adjunctive modafinil/armodafinil in bipolar depression: a meta-analysis of randomized controlled trials. Bipolar Disord.

[12] Agarwal SM, Rao N, Venkatasubramanian G, Behere R, Varambally S, Gangadhar B (2012). Successful use of modafinil in treatment-resistant bipolar depression in an elderly woman. Indian J Psychiatry.

